# Preoperative ketorolac is associated with increased nonunion repair following femoral intramedullary nailing: a retrospective cohort study

**DOI:** 10.1007/s00590-026-04867-y

**Published:** 2026-07-06

**Authors:** Joshua Wang, Carolyn Henein, Cameron Bowers, Philong Nguyen, Keenan Horani, William M. Weiss

**Affiliations:** 1 John Sealy School of Medicine, The University of Texas Medical Branch at Galveston, Galveston, USA; 2 Department of Orthopaedic Surgery and Rehabilitation, The University of Texas Medical Branch at Galveston, Galveston, USA

**Keywords:** Femur, Fracture fixation, Ketorolac, Intramedullary nailing, Outcome assessment, Postoperative complications, Preoperative period

## Abstract

**Purpose:**

Nonsteroidal anti-inflammatory drugs (NSAIDs), including ketorolac, are widely used for perioperative analgesia, but their effects on bone healing remain debated. This study evaluates associations between preoperative intravenous ketorolac and outcomes on bone healing after femoral intramedullary nailing (IMN).

**Methods:**

Using the TriNetX database, we conducted a retrospective cohort analysis of adults undergoing femoral IMN who received a single preoperative IV ketorolac dose. Propensity score matching (1:1) produced 2 cohorts (n = 8,466 each). Outcomes included wound disruption, infection, sepsis, thromboembolism, mortality, readmission, and nonunion/malunion repair. Risk ratios, 95% confidence intervals, and *P* values were calculated (*P* < 0.05).

**Results:**

Ketorolac was associated with increased wound disruption (*P * = 0.034), and nonunion/malunion repair (*P* = 0.015), but decreased mortality (*P* = 0.014) and readmission (*P* = 0.009).

**Conclusion:**

Preoperative ketorolac exposure was associated with higher rates of operative treatment of nonunion/malunion, although these findings should be interpreted cautiously because of substantial residual confounding and exposure misclassification.

**Level of evidence:**

Level III, Prognostic.

**Supplementary Information:**

The online version contains supplementary material available at 10.1007/s00590-026-04867-y.

## Introduction

Intramedullary nailing (IMN) is the gold standard for diaphyseal femoral shaft fractures, providing stable internal fixation via a metal rod within the intramedullary canal, enabling early mobilization and demonstrating superior outcomes compared to plating or external fixation [1, 2]. The technique is associated with high union rates and lower infection risk in closed fractures, but complications, including delayed union, nonunion, malunion, infection, and revision surgery, remain significant concerns, particularly in open fractures or patients with comorbidities [3, 4]. Risk factors affecting IMN outcomes include fracture pattern, surgical technique, timing of fixation, and patient-related factors such as smoking, diabetes, and the use of medications known to interfere with bone metabolism, particularly nonsteroidal anti-inflammatory drugs (NSAIDs) [5].

Ketorolac is a nonselective NSAID commonly administered intravenously for perioperative analgesia to reduce pain and opioid use, acting via inhibition of COX-1 and COX-2–mediated prostaglandin synthesis, which plays roles in both inflammation and bone remodeling and fracture healing [6, 7]. Several clinical studies have suggested that NSAIDs, when used repeatedly or at high doses, may delay fracture healing by impairing osteoblast differentiation and angiogenesis [8]. Some studies report associations between ketorolac use and increased risk of nonunion or delayed union, especially in pediatric or spinal fusion populations, while others, especially those evaluating single doses, report no significant impact on fracture-healing outcomes for femoral and tibial fractures [6].

This study aims to clarify the impact of a single preoperative dose of ketorolac on key postoperative outcomes in femur fracture patients treated with IMN. By examining outcomes up to 2 years postoperatively, this study provides valuable insight into the long-term risks and benefits of ketorolac in orthopedic trauma care.

## Methods

A retrospective cohort analysis was conducted using the TriNetX Research Network, a global federated database of de-identified electronic medical records from 103 healthcare organizations encompassing approximately 105 million patients, with real-time access to demographics, diagnoses, procedures, medications, and laboratory data. All data are de-identified in accordance with the Health Insurance Portability and Accountability Act (HIPAA) and General Data Protection Regulation, qualifying this study as exempt from institutional review board oversight.

### Patient population

Adult patients (≥ 18 years) who underwent IMN of the femur using CPT procedure codes 27,245 and 27,506 between January 1, 2000 and March 20, 2023 were identified. These CPT codes were used as the primary cohort-defining procedure codes because they identify femoral fracture fixation with an intramedullary implant and are not dependent on ICD-10 implementation during the study period. The cohort included patients with femoral fractures treated with intramedullary nailing and was not restricted exclusively to diaphyseal femoral shaft fractures. Therefore, throughout the manuscript, the study population is described as patients with femoral fractures treated with IMN. Because TriNetX relies on coded diagnoses and procedures, granular fracture subtype classification, including shaft, subtrochanteric, or distal femoral fracture patterns, could not be directly adjudicated at the chart or imaging level. Diagnosis, comorbidity, and postoperative outcome variables were identified using available CPT and ICD diagnosis and procedure codes within the TriNetX standardized coding framework. Because the study period spans the transition from ICD-9 to ICD-10, TriNetX-harmonized source data may include legacy diagnosis vocabularies mapped into standardized clinical concepts; however, source-level code harmonization could not be independently adjudicated. Patients were stratified into 2 cohorts: (1) those who received a preoperative dose of intravenous ketorolac on the day of surgery (CPT: 27,245, 27,506) and (2) those who did not receive preoperative ketorolac.

### Demographic characteristics, clinical comorbidities, and outcome variables

Baseline demographic characteristics collected included age, sex, race, and clinical comorbidities assessed prior to surgery (Supplementary Table 1). These variables were included in propensity score matching to ensure balanced comparison groups.

Primary outcomes included surgical treatment of nonunion/malunion assessed within 2 years postoperatively. The specific CPT and ICD-10 codes used for each outcome are provided in Supplementary Table 2. Operative treatment of nonunion/malunion was defined using diagnosis and procedure codes for nonunion, malunion, and related operative repair. Nonunion and malunion were analyzed as a composite outcome because these conditions were captured together within the coding framework. Because outcomes were identified from de-identified EMR coding, the exact operative indication, procedural subtype such as dynamization, exchange nailing, bone grafting, irrigation and debridement, laterality/ipsilateral relationship, and direct relationship to the indexed femoral fracture could not be independently adjudicated at the chart, operative-note, or imaging level. Secondary outcomes included all-cause mortality, hospital readmission, wound disruption, infection (superficial or deep), sepsis, deep vein thrombosis (DVT), pulmonary embolism (PE), and hardware removal. All outcomes were identified using validated CPT and ICD-10 diagnosis and procedure codes available within the TriNetX platform. (Supplementary Table 2).

### Data analyses

Chi-squared tests were used for categorical variables and *t* tests for continuous variables, with statistical significance set at *P* < 0.05. Propensity score matching (1:1 greedy nearest-neighbor method) was used to balance baseline variables between the ketorolac and non-ketorolac groups, minimizing potential confounding. Variables included in the propensity score model were age, sex, race, ethnicity, chronic obstructive pulmonary disease, atherosclerotic heart disease, personal history of nicotine dependence, heart failure, hypertensive disease, chronic kidney disease, nicotine dependence, alcohol-related disorders, hyperlipidemia, type 2 diabetes mellitus, vitamin D deficiency, liver disease, osteoporosis, depressive disorders, opioid use, glucocorticoid use, BMI, and BMI category. Balance between cohorts was assessed using standardized mean differences (SMDs), with an SMD < 0.10 considered indicative of acceptable post-matching balance. SMDs before and after matching are provided in Supplemental Table 1. Analyses were performed using available data within the TriNetX platform, and missing data were not independently imputed because patient-level source data and reasons for missingness were not available. Given the number of postoperative outcomes assessed, statistically significant findings were interpreted cautiously in the context of multiple comparisons. Risk ratios represented cumulative 2-year outcome proportions rather than time-to-event estimates.

**Table 1:** Outcomes After matching comparing preoperative ketorolac users and nonusers

Outcome	Ketorolac use	Control	Risk ratio	95% CI	P value	Absolute risk difference	NNH/NNT
Repair of nonunion/malunion	2.5%	2.0%	1.283	(1.050, 1.568)	0.015	+ 0.5%	NNH = 200
Hospital readmission	6.7%	7.7%	0.866	(0.777, 0.965)	0.009	− 1.0%	NNT = 100
All-cause mortality	12.2%	13.5%	0.906	(0.838, 0.980)	0.014	− 1.3%	NNT = 77
Hardware removal	6.7%	7.3%	0.922	(0.826, 1.029)	0.148	− 0.6%	–
Pulmonary embolism	2.2%	2.1%	1.054	(0.856, 1.299)	0.618	+ 0.1%	–
Deep vein thrombosis	3.3%	3.9%	0.853	(0.726, 1.001)	0.051	− 0.6%	–
Sepsis	8.2%	8.2%	1.001	(0.905, 1.108)	0.978	0.0%	–
Infection	3.8%	3.4%	1.114	(0.953, 1.302)	0.174	+ 0.4%	–
Wound disruption	2.6%	2.1%	1.233	(1.016, 1.498)	0.034	+ 0.5%	NNH = 200

Risk ratios (RRs) with 95% confidence intervals (CIs) and corresponding *P* values were calculated for each postoperative complication. All statistical analyses were performed using the TriNetX Analytics platform (TriNetX, LLC, Cambridge, MA), which employs a combination of SQL, R, and Python for data handling and statistical modeling.

## Results

### Cohort characteristics

Before matching, 8,477 patients received preoperative ketorolac and 43,050 did not. After 1:1 propensity score matching, 8,466 patients remained in each group. Matching resulted in balanced baseline demographics and comorbidities (Supplemental Table 1).

### Postoperative outcomes

Patients receiving preoperative ketorolac experienced significantly higher rates of surgical repair for nonunion/malunion (RR = 1.283; P = 0.015), indicating a potential adverse effect on fracture healing. Additionally, this group showed a higher risk of wound disruption (RR = 1.233; P = 0.034).

**Fig. 1:**
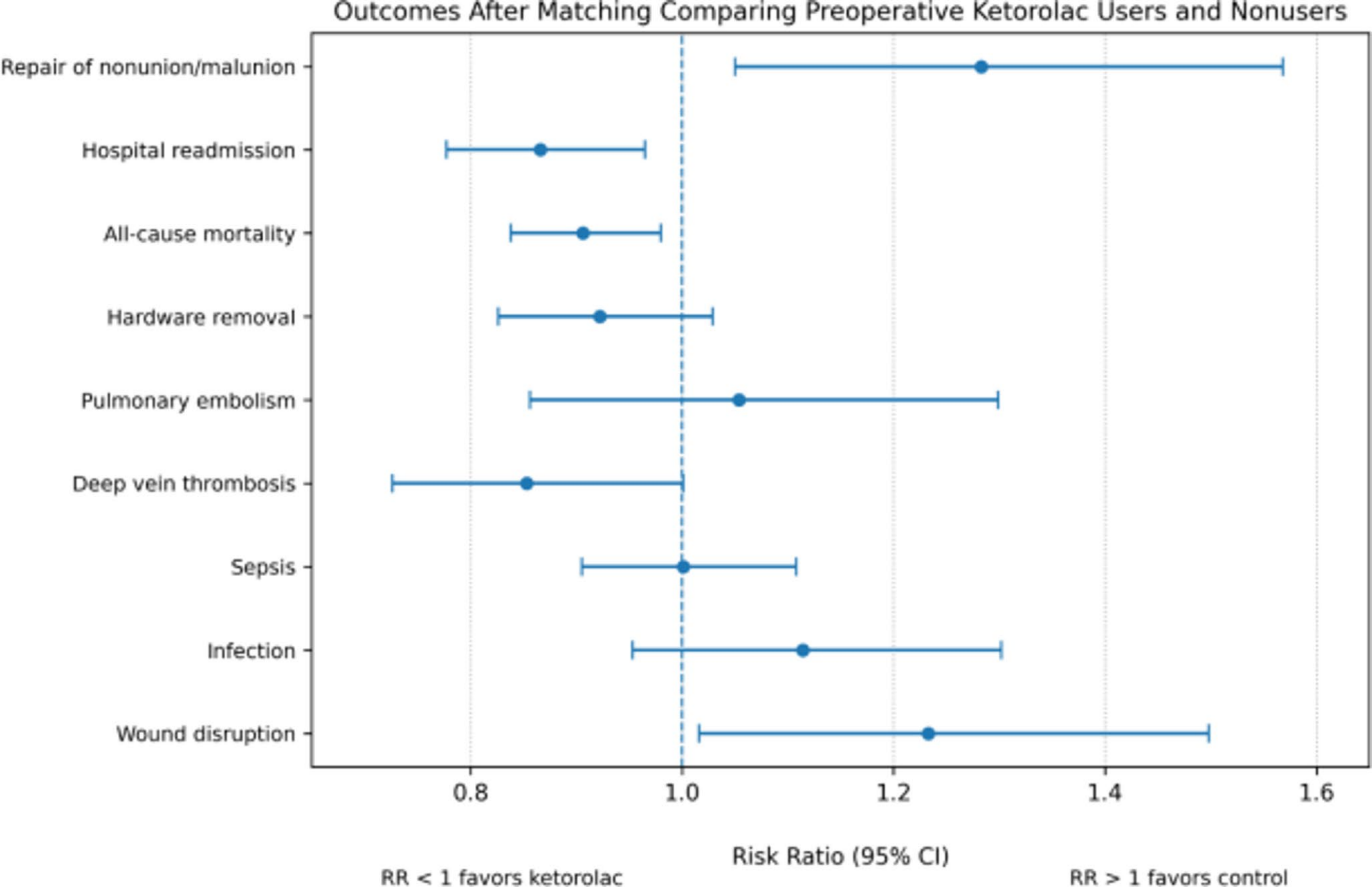
Forest plot showing risk ratio of ketorolac on femoral intramedullary nailing surgical outcomes

Conversely, patients who received ketorolac had lower risks of mortality (RR = 0.906; P = 0.014) and hospital readmission (RR = 0.866; P = 0.009). Rates of sepsis, DVT, PE, infection, and hardware removal did not differ significantly between the 2 groups (Table 1, Fig. 1).

## Discussion

Preoperative ketorolac exposure was associated with higher rates of wound disruption and surgical repair for nonunion/malunion within 2 years after femoral intramedullary nailing. Although ketorolac exposure was also associated with lower rates of all-cause mortality and hospital readmission, these findings are difficult to interpret and should be considered likely non-causal associations. These systemic findings may reflect residual confounding, selection bias, differences in injury severity, perioperative stability, baseline health status, or prescribing patterns that could not be fully captured despite propensity score matching.

Similarly, the increased rate of surgical repair for nonunion/malunion in the ketorolac group reinforces concerns that NSAID administration may interfere with normal osseous healing. COX-2 inhibition reduces inflammatory signaling required for initiating callus formation and can impair the transition from cartilaginous to bony callus, a key step in endochondral ossification [8–10]. Furthermore, models have demonstrated that NSAID use during early fracture healing phases can result in lower callus volume and mechanical strength [7, 11, 12]. This mechanism provides biologic plausibility for the observed association between ketorolac exposure and subsequent surgical correction of impaired union, but causality cannot be inferred from the present study.

Conversely, the lower all-cause mortality observed in patients given preoperative ketorolac should be interpreted cautiously. Given the implausibility that a single documented preoperative ketorolac exposure would independently lower 2-year mortality, a direct mortality benefit from a single documented preoperative ketorolac dose is unlikely. This finding may instead reflect selection bias, residual confounding, or unmeasured differences in injury severity, perioperative stability, prescribing patterns, or baseline health status that were not fully captured despite propensity score matching. Therefore, the observed reduction in mortality should be interpreted as an association rather than evidence that ketorolac independently reduces mortality after femoral intramedullary nailing.

The reduced rate of hospital readmission in the ketorolac group should also be interpreted cautiously and is not sufficient to support a protective effect of preoperative ketorolac. Similar to the mortality finding, the lower readmission rate may reflect residual confounding, differences in perioperative stability, injury severity, discharge disposition, or institutional prescribing practices rather than a direct effect of ketorolac exposure. While several studies have raised concern that prolonged, repeated, or high-dose NSAID exposure may impair fracture healing, other studies evaluating limited perioperative or single-dose ketorolac exposure have not demonstrated a clinically significant increase in nonunion or delayed union [6].

Given the likely non-causal nature of the observed mortality and readmission associations, these findings should be deemphasized when interpreting the clinical implications of preoperative ketorolac exposure. In contrast, the higher rates of operative treatment of nonunion/malunion and wound disruption remain the primary findings of concern. Prior research has consistently demonstrated that NSAID use, particularly during the early phases of bone healing, disrupts normal callus formation and remodeling, potentially leading to long-term complications that require surgical intervention [7, 12]. Given that fracture healing is a biologically demanding process dependent on coordinated inflammatory, proliferative, and remodeling phases, NSAID-mediated prostaglandin inhibition could plausibly contribute to impaired repair in susceptible patients [13]. Emerging alternatives to traditional NSAIDs such as selective prostaglandin E2 receptor (EP4) antagonists are currently under investigation for their ability to provide analgesia without disrupting osseous healing pathways [14]. These agents may offer a promising approach to postoperative pain management with reduced skeletal risk, and thus routine preoperative ketorolac use in femoral IMN should be approached cautiously, particularly in patients at high risk for impaired fracture healing.

Lastly, the elevated risk of wound disruption may reflect ketorolac’s inhibition of prostaglandin-mediated inflammation, which also plays a role in soft tissue healing [15]. Prostaglandins are involved in angiogenesis, fibroblast migration, and collagen deposition. NSAID-mediated suppression of these processes may lead to weaker wound tensile strength and delayed epithelialization, thereby increasing susceptibility to dehiscence or superficial breakdown [16].

### Limitations & strengths

This study has several limitations. As a retrospective electronic medical records-based cohort, residual confounding likely persists despite propensity matching, particularly because several fracture- and treatment-specific variables were unavailable, including fracture type, open versus closed injury status, fracture pattern and severity, surgical technique, timing of fixation, weightbearing protocol, blood loss, and treatment-pathway differences. Although available baseline factors such as smoking- or nicotine-related diagnoses, BMI, comorbidities, opioid use, and glucocorticoid use were included in propensity matching, unmeasured injury severity and clinical management factors may have influenced both ketorolac administration and the risk of nonunion, malunion, or other postoperative complications. Nonunion and malunion were analyzed as a composite outcome because the CPT-based procedural definition captured operative repair of nonunion or malunion together, which reduces clinical interpretability because these are distinct orthopaedic complications. Ketorolac exposure was also limited by the database-based nature of TriNetX, as dose, timing relative to incision, confirmation of true pre-incision administration, intraoperative administration, postoperative ketorolac or other NSAID use, and duration of therapy were unavailable; therefore, exposure misclassification is possible, and the observed associations cannot be attributed to a specific ketorolac dose, timing window, or duration. Coding errors and outcome misclassification are possible, causality cannot be inferred, and outcomes were analyzed as cumulative 2-year event proportions rather than time-to-event endpoints. Future multicenter prospective studies, ideally randomized controlled trials or registry-based studies with chart- and imaging-level adjudication, are needed to clarify whether perioperative ketorolac independently contributes to fracture-healing complications after femoral intramedullary nailing. Despite these limitations, this study has important strengths, including its large sample size, nationally representative database, long-term outcome assessment, and rigorous propensity matching using available baseline characteristics.

## Conclusion

Preoperative ketorolac exposure was associated with higher rates of wound disruption, and operative intervention for nonunion/malunion following femoral IMN. Although ketorolac exposure was also associated with lower rates of mortality and hospital readmission, these findings should be interpreted cautiously as likely non-causal associations related to residual confounding, selection bias, exposure misclassification, and unmeasured differences in injury severity or perioperative stability.

## Supplementary Information

Below is the link to the electronic supplementary material.

Supplementary Material 1

Supplementary Material 2

## Data Availability

No datasets were generated or analysed during the current study.
