# Supplementary material for: Preoperative ketorolac is associated with increased nonunion repair following femoral intramedullary nailing: a retrospective cohort study
Source: Eur J Orthop Surg Traumatol. 2026 Jul 6;36(1):277. doi: 10.1007/s00590-026-04867-y (PMC13337603; doi:10.1007/s00590-026-04867-y)
Supplement: Supplementary file 1 — Supplementary Material 1 [file 590_2026_4867_MOESM1_ESM.docx]

**Supplemental Table 1.** Demographics of Preoperative Ketorolac Users and Nonusers Before and After Propensity Score Matching

| **Variable** | **Cohort** | **Pre-Matched** | | | **Post-Matched** | | |
| --- | --- | --- | --- | --- | --- | --- | --- |
|  |  | **Patients** | **% of Cohort** | ***P*** | **Patients** | **% of Cohort** | ***P*** |
| Age (mean ± SD) | Ketorolac use | 58.0 +/- 20.3 | 100.0% | <0.001 | 58.0 +/- 20.3 | 100.0% | 0.622 |
|  | Control | 59.3 +/- 20.9 | 100.0% |  | 57.8 +/- 20.7 | 100.0% |  |
| White | Ketorolac use | 5,735 | 67.7% | <0.001 | 5,735 | 67.7% | 0.730 |
|  | Control | 30,495 | 71.8% |  | 5,714 | 67.5% |  |
| American Indian or Alaska Native | Ketorolac use | 21 | 0.2% | 0.387 | 21 | 0.2% | 0.763 |
|  | Control | 129 | 0.3% |  | 23 | 0.3% |  |
| Female | Ketorolac use | 4,157 | 49.1% | 0.085 | 4,156 | 49.1% | 0.782 |
|  | Control | 20,410 | 48.1% |  | 4,174 | 49.3% |  |
| Native Hawaiian or Other Pacific Islander | Ketorolac use | 11 | 0.1% | 0.648 | 11 | 0.1% | 0.683 |
|  | Control | 64 | 0.2% |  | 13 | 0.2% |  |
| Not Hispanic or Latino | Ketorolac use | 6,712 | 79.3% | 0.001 | 6,711 | 79.3% | 0.005 |
|  | Control | 32,960 | 77.6% |  | 6,858 | 81.0% |  |
| Hispanic or Latino | Ketorolac use | 455 | 5.4% | <0.001 | 455 | 5.4% | 0.946 |
|  | Control | 2,832 | 6.7% |  | 453 | 5.4% |  |
| Black or African American | Ketorolac use | 1,376 | 16.3% | <0.001 | 1,375 | 16.2% | 0.239 |
|  | Control | 5,772 | 13.6% |  | 1,432 | 16.9% |  |
| Male | Ketorolac use | 3,691 | 43.6% | <0.001 | 3,691 | 43.6% | 0.914 |
|  | Control | 20,170 | 47.5% |  | 3,698 | 43.7% |  |
| Other race | Ketorolac use | 242 | 2.9% | 0.91 | 242 | 2.9% | 0.819 |
|  | Control | 1,223 | 2.9% |  | 247 | 2.9% |  |
| Asian | Ketorolac use | 129 | 1.5% | 0.004 | 129 | 1.5% | 1.000 |
|  | Control | 847 | 2.0% |  | 129 | 1.5% |  |
| Other chronic obstructive pulmonary disease | Ketorolac use | 1,324 | 15.6% | <0.001 | 1,324 | 15.6% | 0.129 |
|  | Control | 5,229 | 12.3% |  | 1,253 | 14.8% |  |
| Atherosclerotic heart disease of native coronary artery | Ketorolac use | 1,335 | 15.8% | 0.947 | 1,335 | 15.8% | 0.202 |
|  | Control | 6,682 | 15.7% |  | 1,275 | 15.1% |  |
| Personal history of nicotine dependence | Ketorolac use | 1,445 | 17.1% | <0.001 | 1,444 | 17.1% | 0.323 |
|  | Control | 5,422 | 12.8% |  | 1,396 | 16.5% |  |
| Heart failure | Ketorolac use | 1,045 | 12.3% | 0.091 | 1,045 | 12.3% | 0.021 |
|  | Control | 5,526 | 13.0% |  | 948 | 11.2% |  |
| Hypertensive diseases | Ketorolac use | 4,197 | 49.6% | <0.001 | 4,196 | 49.6% | 0.113 |
|  | Control | 19,788 | 46.6% |  | 4,093 | 48.3% |  |
| Chronic kidney disease | Ketorolac use | 977 | 11.5% | <0.001 | 977 | 11.5% | 0.059 |
|  | Control | 5,743 | 13.5% |  | 900 | 10.6% |  |
| Nicotine dependence | Ketorolac use | 2,305 | 27.2% | <0.001 | 2,304 | 27.2% | 0.396 |
|  | Control | 7,516 | 17.7% |  | 2,255 | 26.6% |  |
| Alcohol-related disorders | Ketorolac use | 969 | 11.4% | <0.001 | 969 | 11.4% | 0.771 |
|  | Control | 3,429 | 8.1% |  | 957 | 11.3% |  |
| Hyperlipidemia, unspecified | Ketorolac use | 2,646 | 31.3% | <0.001 | 2,646 | 31.3% | 0.310 |
|  | Control | 11,860 | 27.9% |  | 2,585 | 30.5% |  |
| Type 2 diabetes mellitus | Ketorolac use | 1,278 | 15.1% | 0.019 | 1,278 | 15.1% | 0.129 |
|  | Control | 6,842 | 16.1% |  | 1,208 | 14.3% |  |
| Vitamin D deficiency | Ketorolac use | 1,125 | 13.3% | <0.001 | 1,125 | 13.3% | 0.649 |
|  | Control | 4,120 | 9.7% |  | 1,105 | 13.1% |  |
| Diseases of liver | Ketorolac use | 772 | 9.1% | <0.001 | 772 | 9.1% | 0.451 |
|  | Control | 2,884 | 6.8% |  | 744 | 8.8% |  |
| Osteoporosis without current pathological fracture | Ketorolac use | 1,315 | 15.5% | <0.001 | 1,315 | 15.5% | 0.734 |
|  | Control | 5,906 | 13.9% |  | 1,299 | 15.3% |  |
| Depressive episode | Ketorolac use | 1,886 | 22.3% | <0.001 | 1,885 | 22.3% | 0.141 |
|  | Control | 7,128 | 16.8% |  | 1,806 | 21.3% |  |
| Major depressive disorder, recurrent | Ketorolac use | 399 | 4.7% | <0.001 | 398 | 4.7% | 0.770 |
|  | Control | 1,229 | 2.9% |  | 390 | 4.6% |  |
| Opioids | Ketorolac use | 7,052 | 83.3% | <0.001 | 7,051 | 83.3% | 0.951 |
|  | Control | 29,313 | 69.0% |  | 7,048 | 83.3% |  |
| Glucocorticoids | Ketorolac use | 4,003 | 47.3% | <0.001 | 4,002 | 47.3% | 0.242 |
|  | Control | 11,811 | 27.8% |  | 3,926 | 46.4% |  |
| Body mass index | Ketorolac use | 27.0 +/- 7.3 | 61.6% | 0.288 | 27.0 +/- 7.3 | 61.6% | 0.377 |
|  | Control | 26.9 +/- 7.0 | 51.2% |  | 26.9 +/- 7.1 | 59.6% |  |
| 0 - 25 kg/m2 | Ketorolac use | 2,786 | 32.9% | <0.001 | 2,786 | 32.9% | 0.844 |
|  | Control | 11,632 | 27.4% |  | 2,774 | 32.8% |  |
| 25 - 30 kg/m2 | Ketorolac use | 2,323 | 27.4% | <0.001 | 2,322 | 27.4% | 0.617 |
|  | Control | 9,577 | 22.6% |  | 2,293 | 27.1% |  |
| 35 - 40 kg/m2 | Ketorolac use | 676 | 8.0% | <0.001 | 675 | 8.0% | 0.105 |
|  | Control | 2,545 | 6.0% |  | 619 | 7.3% |  |
| 40 - 100 kg/m2 | Ketorolac use | 476 | 5.6% | <0.001 | 475 | 5.6% | 0.343 |
|  | Control | 1,705 | 4.0% |  | 447 | 5.3% |  |
