# Supplementary material for: Preoperative ketorolac is associated with increased nonunion repair following femoral intramedullary nailing: a retrospective cohort study
Source: Eur J Orthop Surg Traumatol. 2026 Jul 6;36(1):277. doi: 10.1007/s00590-026-04867-y (PMC13337603; doi:10.1007/s00590-026-04867-y)
Supplement: Supplementary file 2 — Supplementary Material 2 [file 590_2026_4867_MOESM2_ESM.docx]

| Supplementary Table 2: Codes Used to Define Covariates and Outcomes | | |
| --- | --- | --- |
| Variable | **Code Type** | **Code(s)** |
| Chronic obstructive pulmonary disease | ICD-10-CM | J44 |
| Atherosclerotic heart disease | ICD-10-CM | I25.1 |
| Personal history of nicotine dependence | ICD-10-CM | Z87.891 |
| Heart failure | ICD-10-CM | I50 |
| Hypertensive diseases | ICD-10-CM | I10-I1A |
| Chronic kidney disease | ICD-10-CM | N18 |
| Nicotine dependence | ICD-10-CM | F17 |
| Alcohol-related disorders | ICD-10-CM | F10 |
| Hyperlipidemia | ICD-10-CM | E78.5 |
| Type 2 diabetes mellitus | ICD-10-CM | E11 |
| Vitamin D deficiency | ICD-10-CM | E55 |
| Diseases of liver | ICD-10-CM | K70-K77 |
| Osteoporosis without current pathological fracture | ICD-10-CM | M81 |
| Depressive episode | ICD-10-CM | F32 |
| Major depressive disorder, recurrent | ICD-10-CM | F33 |
| Opioids | ATC/Medication class | N02A |
| Glucocorticoids | ATC/Medication class | H02AB |
| Outcome |  |  |
| Repair of nonunion/malunion | CPT | 1005087 |
| Hospital readmission | ICD-10-CM | Z48.89 |
| All-cause mortality | Demographic variable | Deceased |
| Hardware removal | CPT | 20680; 20670 |
| Pulmonary embolism | ICD-10-CM | I26 |
| Deep vein thrombosis | ICD-10-CM | I82.4 |
| Sepsis | ICD-10-CM | A41.9 |
| Infection | ICD-10-CM | T81.4 |
| Wound disruption | ICD-10-CM | T81.3 |
